# Commentary: The absence of protein Y4yS affects negatively the abundance of T3SS *Mesorhizobium loti* secretin, RhcC2, in bacterial membranes

**DOI:** 10.3389/fmicb.2015.00710

**Published:** 2015-07-14

**Authors:** Anastasia D. Gazi

**Affiliations:** Laboratoire d'Enzymologie et Biochimie Structurales, Centre National de la Recherche ScientifiqueGif-sur-Yvette, France

**Keywords:** secretin, pilotin, rhizobia, type III secretion system, type II secretion system, tight adherence secreton

Bacteria secrete macromolecules to manipulate their surrounding environment (abiotic or biotic one) for their own benefit. Among them, the diderms (Gram-negative), face the challenge of protein transport across an additional, important barrier: the outer membrane (OM). Specific pathways have evolved to transfer lipidated polypeptide chains to the OM, for example, the Lol system (Okuda and Tokuda, 2011). These polypeptides are then inserted to the inner OM leaflet via their N-terminal-attached lipid moiety. Some of them adopt a β-barrel fold or polymerize in a specific way to accomplish their complete insertion in the membrane. A large family of protein domains, named secretins (Figure 1; Korotkov et al., 2011), that polymerize to form a channel in the OM, is present in many bacterial secretion systems, including the Type II Secretion System (T2SS), the Type IV pili (both T4aP and T4bP types) and the Type III Secretion System (T3SS).

**Figure 1:**
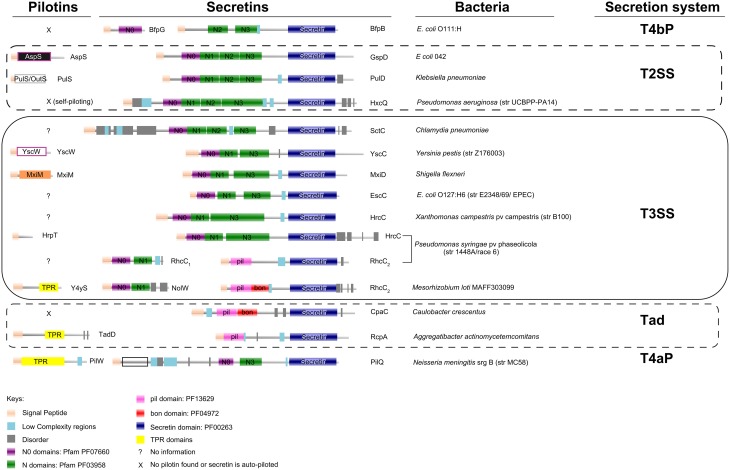
**Protein domain organization of secretins and pilotins from Type II, Type III Secretion Systems, Type IV pili and the Tad system**. Secretins of the *Rhizobium* (*Rhc-*) family of T3SSs, represented here from subgroup I *Rhc-*T3SS of *Mesorhizobium loti* and subgroup II *Rhc*-T3SS of *Pseudomonas syringae* pv phaseolicola (rows 12 and 11 respectively), are encoded by two different genes. The Rhc2 polypeptides are obviously related to the Tad system (rows 13 and 14) rather than to secretins from other T3SS families (rows 5–10). Pilotins (column 1) do not share common domains and seem to adapt very diverse folds. The domain organization analysis was based on Pfam database (Finn et al., 2014; http://pfam.xfam.org/), complemented by SMART (Letunic et al., 2015; http://smart.embl-heidelberg.de/). More than half of the N0 domains present were recognized through threading (Kelley and Sternberg, 2009; http://www.sbg.bio.ic.ac.uk/phyre2).

In a generalized scheme, the nascent polypeptide of a secretin carries an N-terminal signal that is recognized by the general secretion pathway (Sec). The Sec system transfers the polypeptide to the periplasmic space. Once there, most often an escorting lipoprotein, pilotin, or docking protein that is also targeted to the periplasmic space via the Sec or Tat pathways will assist with delivery, insertion and/or right polymerization of the polypeptide in the OM, most likely through enrollment of the Lol system (Collin et al., 2011; Okuda and Tokuda, 2011).

Pilotins are extremely diverse in their folding and way of action, while they are usually specific to their cognate secretins (Figure 1; Koo et al., 2012; Dunstan et al., 2013). In most cases, they are encoded within gene clusters that also encode their cognate secretins, as well as other subunits of the respective bacterial secretion system they serve.

Type III secretion systems evolved for the direct translocation of bacterial proteins from the bacterial cytoplasm to the eukaryotic cell cytoplasm (Soto et al., 2006; Tampakaki et al., 2010; Marteyn et al., 2012; Tampakaki, 2014), a process involved in either pathogenesis or symbiosis with higher organisms. These systems appear to have evolved from the bacterial flagellum through gene loss and new gene acquisitions. Several modern T3SS families exist today that are specialized in the invasion of a broad range of hosts, animals or plants (Abby and Rocha, 2012).

The bacterial flagellum does not possess a secretin while a T2SS/T4P originated secretin has been early acquired and evolved to a specialized T3SS secretin. However, in the case of the T3SS of *Rhizobia* (*Rhc*-T3SS), dedicated to symbiosis, this early acquired T2SS/T4P related secretin was lost and only a shorter aminoterminal remnant of this gene (named *rhcC1*), is present in the T3SS gene cluster (Figure 1), while an acquisition of a Tad (Tight Adherence)—like secretin (named *rhcC2*) took place, possibly through DNA recombination events (Figure 1; Abby and Rocha, 2012).

The work of Mercante et al. (2015) nicely complements these findings by showing that a predicted lipoprotein (*y4yS*) of the *Mesorhizobium loti Rhc*-T3SS could act as a potential pilotin by influencing the level of RhcC2 protein present in bacterial membranes, while sharing common characteristics (tetatricopeptide repeats, TPRs) of the Tad system pilotins.

Surprisingly, TPR domains have not been observed in other known pilotins within the remaining T3SS families, while they are present in Tad and T4aP pilotins (Figure 1). This new finding, raised new questions. The mechanism by which Y4yS is piloting the *Rhc*-secretin to the OM still remains unknown. Furthermore, *y4yS* homologs are only present in a subset of *Rhc*-T3SSs (Gazi et al., 2012). *Rhc*-T3SS subgroup II clusters do not harbor a *y4yS* gene, while *Rhc*-T3SS subgroup III of *Rhizobium etli* although maintaining a *rhcC1* gene, seems to have lost or never acquired a Tad like secretin (*rhcC2*), adding even more mystery to how this particular T3SS nanomachine transverses the OM. Why is a system-specific, non-conserved pilotin needed for the delivery and accommodation of a highly conserved domain in the outer bacterial membrane? This question cannot be answered as of this time, but it seems quite possible that more studies will further reveal this complexity before giving us some first clues.

## Conflict of interest statement

The author declares that the research was conducted in the absence of any commercial or financial relationships that could be construed as a potential conflict of interest.
